# Severe Histoplasmosis in Travelers to Nicaragua

**DOI:** 10.3201/eid0910.030049

**Published:** 2003-10

**Authors:** Michelle Weinberg, Julia Weeks, Susan Lance-Parker, Marc Traeger, Steven Wiersma, Quyen Phan, David Dennison, Pia MacDonald, Mark Lindsley, Jeannette Guarner, Patricia Connolly, Martin Cetron, Rana Hajjeh

**Affiliations:** * Centers for Disease Control and Prevention, Atlanta, Georgia, USA; † Archbold Urgent Care Center, Thomasville, Georgia, USA; ‡ Georgia Department of Human Resources, Division of Public Health, Atlanta, Georgia, USA; § Florida Department of Health, Tallahassee, Florida, USA; ¶ Connecticut Department of Public Health, Hartford, Connecticut, USA; # Highlands-Cashiers Hospital, Highlands, North Carolina, USA; ** North Carolina Division of Public Health, Raleigh, North Carolina, USA; †† Indiana University School of Medicine and Histoplasmosis Reference Laboratory, Indianapolis, Indiana, USA

## Abstract

We investigated an outbreak of unexpectedly severe histoplasmosis among 14 healthy adventure travelers from the United States who visited a bat-infested cave in Nicaragua. Although histoplasmosis has rarely been reported to cause serious illness among travelers, this outbreak demonstrates that cases may be severe among travelers, even young, healthy persons.

Histoplasmosis is a systemic infection caused by the dimorphic fungus, *Histoplasma capsulatum*. Infection results from inhaling spores, usually through exposure to bat and bird droppings in barnyards and caves. Although outbreaks have occurred after visits to bat-infested caves, histoplasmosis has not been frequently recognized as travel-related and has rarely led to serious illness among young, healthy travelers. This fungus is endemic in the United States along the Ohio and Mississippi River valleys and many other parts of the world, particularly Latin America. Histoplasmosis is often asymptomatic in endemic settings, but infection can result in a spectrum of illness, ranging from mild influenzalike illness to acute pulmonary infection and disseminated extrapulmonary disease. Immunocompromised persons and the elderly are at greater risk for disseminated disease (*1*). This report describes a recent outbreak of histoplasmosis among U.S. adventure travelers to Nicaragua that was associated with a high attack rate and hospitalizations among previously healthy travelers.

## The Study

In June 2001, five persons with a febrile respiratory illness visited a community hospital. The patients were among 15 persons (age range 19–61 years; median 38 years), residents of four states, who had participated in a geology-biology community college class trip to Nicaragua. To determine the cause and describe characteristics of the outbreak, we interviewed trip participants and reviewed medical records to collect demographic information, clinical history, and activities during the trip. Based on the partcipant’s exposure to a cave with bats and clinical characteristics suggestive of acute histoplasmosis, diagnostic testing for histoplasmosis was conducted.

A case of acute histoplasmosis was defined as illness in a person who tested positive by one of the following laboratory tests: serology, urine antigen, histopathology, or culture. Immunodiffusion and complement- fixation serologic tests were performed at the Centers for Disease Control and Prevention (CDC). The immunodiffusion test was considered positive if an H or M band or both were detected. A complement-fixation titer to the yeast or mycelial antigens of >1:32 or a fourfold increase in the titer between acute- and convalescent-phase serum specimens was considered evidence of acute infection. Acute-phase serum specimens were obtained 2–3 weeks after exposure. Convalescent-phase serum specimens were collected approximately 3 months after exposure. One patient underwent bronchoalveolar lavage and bronchoscopy, and a biopsy specimen was obtained. Lung tissue was stained with hematoxylin and eosin and Gomori methenamine silver stains. Bronchoalveolar lavage washings were cultured by conventional methods (*2*). Identification of the organism as *H. capsulatum* was confirmed by GenProbe assay (GenProbe, San Diego, CA). Urine antigen tests, which were performed 2 weeks after exposure, are considered positive if >1 unit is detected (*3*,*4*).

The travelers began their trip on May 19 and returned to the United States on May 30. During their trip, they explored rock quarries, visited a biological research station, swam in freshwater lakes, and were exposed to parrots and insects. On May 21, a total of 14 of the 15 travelers entered a small cave that had previously been used as a silver mine. They remained in the cave for 10 minutes, during which time they saw several flying bats and bat guano on the ground.

The infection rate was 100% among the 14 travelers who entered the cave, and 12 (86%) were symptomatic. All 14 patients had serologically confirmed infection. Of the 14 patients, 12 (86%) had urine antigen tests (Table). The only traveler who did not enter the cave tested negative by serology and urine antigen.

**Table:** Summary of laboratory testing results and clinical outcomes among persons with histoplasmosis

Characteristic	N (%)
Diagnostic test result^a^	
CF^b^ and ID^c^ positive	5 (36)
CF positive, ID negative	8 (57)
ID positive, CF negative	1 (7)
Urine antigen positive^d^	7 (58), range 1.2–4.6, median 2.8
Clinical outcomes^e^	
Missed work, school, or both^f^	10 (83)
Treated with itraconazole	9 (75)
Treated with steroids	7 (58)
Hospitalized	6 (50)
Duration of hospitalization	Median 6 d (range 2–11 d)
Duration of fever	Median 12 d (range 4–26 d)
Duration of symptoms	Median 42 d (range 16–210 d)

^a^Among 14 persons with histoplasmosis. ^b^CF: complement fixation. ^c^ID: immunodiffusion. ^d^Urine antigen tests were performed for 12 persons. ^e^Among the 12 symptomatic persons. ^f^Five persons missed 2 weeks–3 months of work, and five persons missed one semester of school.

Of the infected travelers, 8 (57%) were female. The average incubation period was 11 days (range 10–12 days), assuming that exposure occurred on May 21. Among the 12 symptomatic persons, 12 (100%) had a fever of 38.9°C to 40°C, nonproductive cough, myalgias, fever, chills, night sweats, loss of appetite, and mild headache. Many had nausea and vomiting (83%), chest pain (58%), and arthralgias (58%). Six persons had mild to moderate respiratory distress, with oxygen saturation of 88% to 95%. On physical examination, all the travelers’ lung fields were clear to auscultation, a feature consistent with histoplasmosis infection. The two asymptomatic persons had previous exposure to bats or caving. Among the symptomatic persons, we found no differences in severity of symptoms according to sex, prior exposure to spelunking, residence in a histoplamosis-endemic area, or activities while in the mine.

In accordance with clinical treatment guidelines, physicians decided to treat patients with itraconazole for 6 to 12 weeks; some patients also received steroids (*5*). No patients required discontinuation of therapy was discontinued because of adverse effects.

Laboratory studies showed normal complete blood counts, serum chemistries, and renal function tests. Four persons had mild to moderate elevation in liver function tests (alanine aminotransferase, range 55–204; aspartate aminotransferase, range 48–153; and alkaline phosphatase, range 95–311). All 12 symptomatic persons had abnormal chest radiograph results with bilateral nodular infiltrates (Figure). Two persons had hilar adenopathy, and one had a small pleural effusion.

**Figure:**
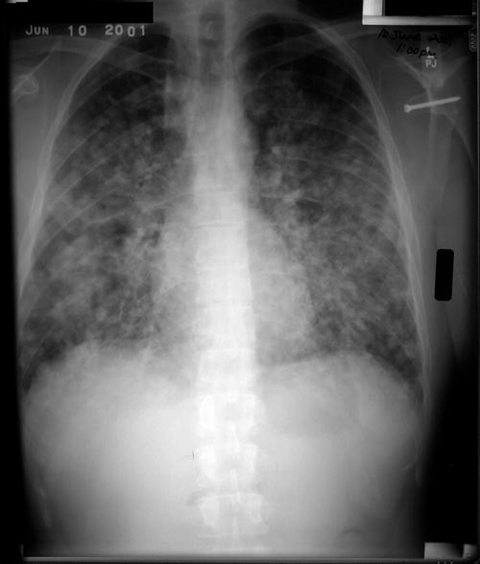
Chest radiograph of patient who acquired acute pulmonary histoplasmosis after visiting a cave in Nicaragua.

Hematoxylin and eosin–stained sections of the lung tissue from the biopsy showed macrophages and neutrophils in the interstitium; no granulomatous inflammation was observed. Gomori methenamine silver stain demonstrated budding yeasts compatible with *H. capsulatum*. Culture of the bronchoalveolar lavage fluid from this patient grew *H. capsulatum* after 4 weeks of incubation.

## Conclusions

This report highlights the importance of histoplasmosis as a potentially serious travel-related illness. Histoplasmosis is usually considered a mild, self-limited illness; however, this outbreak of histoplasmosis among previously healthy travelers was associated with a 100% infection rate of histoplasmosis and an 86% rate of symptomatic infection in the persons who entered the cave. Among the symptomatic persons, 50% required hospitalization and 83% missed school or work (range: 2 weeks–3 months) as a result of their illness (Table). Because of the high attack rate and the large percentage of patients with severe infection, these travelers were likely exposed to very high concentrations of *H. capsulatum* spores during their brief visit inside the cave.

*H. capsulatum* exists throughout the world (*1*,*6*); however, nonimmune travelers from areas with a low prevalence of histoplasmosis, who engage in high-risk activities in disease-endemic areas, are at greater risk of acquiring symptomatic fungal infection. The number of outbreaks of histoplasmosis, especially among U.S. travelers to Latin America, has increased. Histoplasmosis has been reported in groups of travelers who entered caves with bats in Costa Rica (*7*; unpub. data, Centers for Disease Control and Prevention CDC), Ecuador (*8*), and Peru (*9*). In 2001, more than 200 college students became infected with histoplasmosis during a spring break trip to Acapulco, Mexico (*10*,*11*). Outbreaks and isolated cases of histoplasmosis have also been described among non–-U.S. travelers to Latin America and other areas of the world (*12*–*19*).

Clinicians should consider fungal pathogens when evaluating returning travelers who have a febrile respiratory syndrome. The differential diagnosis of acute febrile respiratory illness in international adventure travelers is extensive and includes legionellosis, psittacosis, leptospirosis, schistosomiasis, histoplasmosis, coccidioidomycosis, influenza, parainfluenza, mycoplasma, dengue, and malaria.

The timely diagnosis of histoplasmosis in travelers may be particularly challenging for clinicians evaluating one sporadic case or a patient involved in an undetected multistate outbreak. Antibody response can take 4–6 weeks to develop, reducing its usefulness in the acute setting and requiring the collection of a convalescent-phase specimen for confirmation. Antibodies may also persist for 5 years, making it difficult to distinguish between prior and recent infection without obtaining a convalescent-phase specimen. The sensitivity of immunodiffusion is 72% to 85%, while that of complement fixation is 80% to 90% (*20*,*21*). The antigen test can be used for urine, cerebrospinal fluid, serum, and bronchoalveolar lavage fluid, and results are available in 24 hours. This test had been used most extensively in AIDS patients; however, it is less sensitive in nonimmunocompromised persons with acute pulmonary infection. Sensitivity of the urine antigen test for detection of acute pulmonary infection ranges from 44% to 75% and increases when performed early after the onset of symptoms (*3*,*4*).

In this outbreak, 80% of the travelers were prescribed appropriate malaria chemoprophylaxis during a pretravel assessment. However, most clinicians may not have thought to counsel them about histoplasmosis risk and prevention. During pretravel visits, clinicians should review patients’ itineraries for possible fungal exposures and should counsel them to avoid entering caves, especially those known to be bat infested. Since travelers may not know whether their itinerary includes visits to bat-infested caves, adventure travelers and persons who are at high risk for severe histoplasmosis infection, such as immunocompromised travelers (especially persons with AIDS), should be counseled to avoid possible exposures, either by avoiding caves or by using special protective masks. The National Institute for Occupational Safety and Health has published guidelines for special masks that can be used to reduce occupational and other exposures (e.g., among spelunkers). Unfortunately, these special masks are bulky and may be impractical for many travelers (*22*). While most nonimmunocompromised patients with acute pulmonary histoplasmosis improve without treatment, persons with hypoxemia, diffuse pulmonary histoplasmosis, or severe illness requiring hospitalization may benefit from antifungal therapy and, in some cases, corticosteroids (*5*).

The contribution of histoplasmosis to infectious disease illness in travelers is likely underestimated. As adventure travel becomes increasingly accessible, especially among persons at high risk, immunocompromised persons, and the elderly, histoplasmosis and other fungal infections in travelers may become more common. Enhanced surveillance for histoplasmosis and other fungal infections by public health officials, combined with heightened awareness by clinicians who are evaluating symptomatic, post-travel patients, can lead to greater understanding of the epidemiology of fungal infections among travelers and, ultimately, to improved prevention measures.

## References

[1] Deepe G. *Histoplasma capsulatum.* In: Mandel GL, Bennett JE, Dolin R, editors. Principles and practice of infectious diseases. 5th ed. New York: Churchill Livingston; 2000. p. 2718–33.

[2] Larone DH, Mitchell TG, Walsh TJ. Histoplasma, blastomyces, coccidioides, and other dimorphic fungi causing systemic mycoses. In: Murray PR, editor. Manual of clinical microbiology. 7th ed. Washington: ASM Press; 1999. p. 1259–68.

[3] Williams B, Fojtasek M, Connolly-Stringfield P, Wheat J. Diagnosis of histoplasmosis by antigen detection during an outbreak in Indianapolis, Ind. Arch Pathol Lab Med. 1994;118:1205–8. 7979915

[4] Wheat LJ, Kohler RB, Tewari RP. Diagnosis of disseminated histoplasmosis by detection of *Histoplasma capsulatum* antigen in serum and urine specimens. N Engl J Med. 1986;314:83–8. 10.1056/NEJM198601093140205 3941695

[5] Wheat J, Sarosi G, McKinsey D, Hamill R, Bradsher R, Johnson P, Practice guidelines for the management of patients with histoplasmosis. Clin Infect Dis. 2000;30:688–95. 10.1086/313752 10770731

[6] Wilson ME. A world guide to infections: diseases, distribution, diagnosis. Oxford: Oxford University Press; 1991. p.190.

[7] Centers for Disease Control and Prevention. Cave-associated histoplasmosis—Costa Rica. MMWR Morb Mortal Wkly Rep. 1988;37:312–3. 3130563

[8] Valdez H, Salata RA. Bat-associated histoplasmosis in returning travelers: case presentation and description of a cluster. J Travel Med. 1999;6:258–60. 10.1111/j.1708-8305.1999.tb00529.x 10575176

[9] Nasta P, Donisi A, Cattane A, Chiodera A, Cassari S. Acute histoplasmosis in spelunkers returning from Mato Grosso, Peru. J Travel Med. 1997;4:176–8. 10.1111/j.1708-8305.1997.tb00815.x 9815510

[10] Centers for Disease Control and Prevention. Outbreak of acute respiratory febrile illness among college students—Acapulco, Mexico, March 2001. MMWR Morb Mortal Wkly Rep. 2001;50:261–2. 11411829

[11] Centers for Disease Control and Prevention. Update: outbreak of acute febrile respiratory illness among college students—Acapulco, Mexico, March 2001. MMWR Morb Mortal Wkly Rep. 2001;50:359–60. 11465910

[12] Bonnet D, Balandraud P, Lonjon T, Rey P, Van de Walle JP, Cador L, Round pulmonary lesions after returning from French Guyana. Six cases of American pulmonary histoplasmosis. Med Trop. 1995;55:55–60. 7637611

[13] Buxton JA, Dawar M, Wheat LJ, Black WA, Ames NG, Mugford M, Outbreak of histoplasmosis in a school party that visited a cave in Belize: role of antigen testing in diagnosis. J Travel Med. 2002;9:48–50. 10.2310/7060.2002.22444 11953263

[14] Erkens K, Lademann M, Tintelnot K, Lafrenz M, Kaben U, Reisinger EC. Histoplasmosis group disease in bat researchers returning from Cuba. Dtsch Med Wochenschr. 2002;127:21–5. 10.1055/s-2002-19428 11905225

[15] Fujio J, Nishimura K, Miyaji M. Epidemiological survey of the imported mycoses in Japan. Nippon Ishinkin Gakkai Zasshi. 1999;40:103–9. 10.3314/jjmm.40.103 10234082

[16] Gascon J, Torres JM, Luburich P, Ayuso JR, Xaubet A, Corachan M. Imported histoplasmosis in Spain. J Travel Med. 2000;7:89–91. 10.2310/7060.2000.00028 10759576

[17] Hatakeyama S, Kashiyama T, Takechi A, Sasaki S, Akamatsu E. Cave-associated acute pulmonary histoplasmosis in two Japanese returning from Mexico. Nihon Kokyuki Gakkai Zasshi. 2001;39:293–7. 11481831

[18] Hirsch D, Leupold W, Rupprecht E. Pulmonary histoplasmoma after travel abroad. Pneumologie. 1996;50:242–4. 8919921

[19] Suzaki A, Kimura M, Kimura S, Shimada K, Miyaji M, Kaufman L. An outbreak of acute pulmonary histoplasmosis among travelers to a bat-inhabited cave in Brazil. Kansenshogaku Zasshi. 1995;69:444–9. 7751754 10.11150/kansenshogakuzasshi1970.69.444

[20] Morrison CJ, Lindsley MD. Serological approaches to the diagnosis of invasive fungal infections. In: Calderone R, Cihlar R, editors. Fungal pathogenesis: principles and practice. New York: Marcel Dekker; 2001. p. 667–716.

[21] Reiss E, Kaufman L, Kovacs JA, Lindsley MD. Clinical immunomycology. In: Rose NR, Hamilton RG, Detrick B, editors. Manual of clinical laboratory immunology. Sixth edition. Washington: American Society for Microbiology; 2002. p. 559–83.

[22] Centers for Disease Control and Prevention. National Institute for Occupational Safety and Health. Histoplasmosis: protecting workers at risk, revised guidelines for preventing histoplasmosis; 1997. Available from: URL: http://www.cdc.gov/niosh/97-146.html

